# Detection of a divergent Parainfluenza 4 virus in an adult patient with influenza like illness using next-generation sequencing

**DOI:** 10.1186/1471-2334-14-275

**Published:** 2014-05-19

**Authors:** Seweryn Bialasiewicz, Jodie McVernon, Terry Nolan, Stephen B Lambert, Guoyan Zhao, David Wang, Michael D Nissen, Theo P Sloots

**Affiliations:** 1 Queensland Children’s Medical Research Institute, The University of Queensland, Brisbane, Qld, Australia; 2 Queensland Paediatric Infectious Diseases Laboratory, The Royal Children’s Hospital, Brisbane, Qld, Australia; 3 Murdoch Children’s Research Institute & Melbourne School of Population Health, The University of Melbourne, Parkville, Vic, Australia; 4 Departments of Molecular Microbiology and Pathology & Immunology, Washington University School of Medicine, St. Louis, MO, USA; 5 Sir Albert Sakzewski Virus Research Centre, Building C28, Back Rd, Herston, QLD 4029, Australia

**Keywords:** Parainfluenza 4, Community infection, Respiratory tract infection, Influenza like illness, Next generation sequencing, Virus discovery, PCR, False negative results, Adult

## Abstract

**Background:**

Human Parainfluenza viruses are a common cause of both upper and lower respiratory tract infections, particularly in children. Of the four Parainfluenza virus serotypes, Parainfluenza 4 is least well characterised from both the clinical, epidemiological and genetic perspectives.

**Methods:**

Flocked nose or throat swabs from a previous study investigating viral prevalence in community-based adults suffering from influenza like illness were used as the basis for this study. Samples in which no virus was detected using a 16 viral respiratory pathogen real-time PCR panel were barcoded and pyrosequenced using the Roche 454 GS FLX Titanium chemistry. The sequences were analysed using the VirusHunter bioinformatic pipeline. Sanger sequencing was used to complete the detected Parainfluenza 4 coding region.

**Results:**

A variant Parainfluenza 4 subtype b strain (QLD-01) was discovered in an otherwise healthy adult who presented with influenza like illness. Strain QLD-01 shared genomic similarities with both a and b subtypes. The extent of divergence of this genome from the 5 available whole Parainfluenza 4 genomes impacted the predicted binding efficiencies of the majority of published Parainfluenza 4 PCR assays.

**Conclusions:**

These findings further support a possible role for Parainfluenza 4 in the aetiology of adult respiratory disease within the community setting, and highlight the caution needed to be used in designing PCR assays from limited sequence information or in using proprietary commercial PCR assays.

## Background

Human parainfluenza viruses are a common cause of both upper and lower respiratory tract infections, particularly in children [1-5]. Four serotypes are known, but most epidemiological and clinical research has been focused on parainfluenza serotypes 1–3. This has been primarily due to the poor growth characteristics in cell culture of parainfluenza 4 (PIV4), the lack of commercial diagnostic reagents, and historical exclusion from routine diagnostic testing [3]. Two antigenically distinct PIV4 subtypes, PIV4a and PIV4b, exist [6]. Functionally and epidemiologically, little is known about the two PIV4 subtypes, however both are capable of co-circulating within the same population [7]. With the advent of reverse transcription PCR (RT-PCR), it has become easier to screen for an expanded range of RNA viruses, leading to a re-examination of PIV4’s epidemiology and role in human disease [2,3,5,8]. The majority of research into PIV4 has focused on children within the hospital setting, however little information is available on the role of PIV4 in disease within the broader community.

Despite the resurgent interest in PIV4, a dearth of publically available PIV4 sequences, and in particular, whole genomes, still exists. The lack of appreciable sequence information hampers the design and evaluation of sensitive research and diagnostic RT-PCR assays, since these tests are reliant on oligomer homology to the target sequence. Thus it is imperative to increase the number of publically available sequences for clinically relevant pathogens where little information is currently available, particularly when variant genomes are observed.

Powerful new techniques such as next-generation sequencing have been applied to clinical samples over the last five years with the aims of discovering novel pathogens. Numerous viruses and variant strains have been identified using this approach, including a divergent PIV4 subtype a isolate late in 2013 [9]. Unlike insensitive traditional virological methods and highly specific RT-PCR, next-generation sequencing methods have the advantage of being able to sequence total or targeted DNA and RNA from samples in an unbiased way, without *a priori* knowledge of the possible viral agent(s) present, thus making them the ideal tool for novel and divergent viral genome discovery.

In this study, we used a combination of RT-PCR and next-generation sequencing to identify and characterise the full coding sequence of a novel PIV4 variant from an adult participating in a community-based cohort study of respiratory illness. Furthermore we compared the primer sequences of existing RT-PCR assays to the genome of this variant strain.

## Methods

### Respiratory samples

Samples used for this study were derived from a previous study [10] investigating viral prevalence in a sub-population of participants in a community-based, randomised control trial assessing influenza vaccine effectiveness. Briefly, flocked nose or throat swabs were collected from otherwise healthy adults aged from 18–64 years who presented with influenza-like illness (ILI). ILI was defined as cough, sore throat, runny nose or nasal congestion and at least one systemic symptom (fever greater/equal than 37.8°C, feverishness, chills or myalgia). In total, 643 samples were screened for adenovirus, human metapneumovirus, parainfluenza viruses 1, 2 & 3, respiratory syncytial virus, influenza A and B, picornaviruses, bocavirus, coronaviruses (OC43, 229E, NL63 and HKU1) and WU and KI polyomaviruses using real-time PCR [10]. 299 study samples from which no viral pathogen was detected were used as templates for novel virus discovery. Written consent was obtained from all study participants. The original and current studies were approved by the Royal Children’s Hospital Human Research Ethics Committee (Melbourne) and the Human Research Protection office of Washington University, respectively.

### Viral discovery pipeline

Total nucleic acid was extracted from each sample, subjected to sample-specific barcoded random-priming cDNA synthesis and then PCR amplified using barcode-specific primers. Standard library construction and 454 GS FLX Titanium pyrosequencing was performed as previously described [11]. The sequences were analysed using the VirusHunter bioinformatic pipeline [12]. In brief, high quality reads with similarity to viruses at the nucleotide level or amino acid level were identified using BlastN and BlastX, respectively.

### Genome sequencing & assembly

Individual 454 reads were assembled and mapped against PIV4 subtype a and PIV4 subtype b reference genomes M-25 (AB543336) and SKPIV4 (EU627591), respectively. Walking primers for amplifying and sequencing the remainder of the genome were designed based on the assembled contigs and reference genomes (see Additional file 1). Sanger sequencing was performed on the overlapping amplified cDNA bidirectionally. Contig assembly and genome characterisation was performed using CLC Bio Genomics Workbench 6.5 software. (CLC Bio, Denmark) Phylogenetic analysis was performed using MEGA 5.2 software (http://www.megasoftware.net/) [13]. Recombination event analyses were performed using the Recombination Analyses Tool software (https://github.com/ethering/RAT) [14], with widow sizes of 1719, 800, and 400 applied to a whole genome alignment using QLD-01 as the reference.

## Results

A sample collected in Melbourne (Australia) from a 49 year old male produced 32 reads (see Additional file 2) with highest similarity to PIV4 (83.7-98.3%). Apart from the presentation with ILI meeting the study case definition, no other clinical information was recorded in the subject’s symptom diary. The reads mapped to six singletons and five contigs of 533, 607, 700, 759, and 904 nt in length, and showed highest similarity to SKPIV4, against which the genome walking primers were designed. Final assembly of the sequenced amplicons yielded a near-complete genome of 17,160 nt in length, and included the entire coding region. The sequence was submitted to GenBank as isolate QLD-01 (KF908238). During the study period, one other PIV4 detection was observed within the study population.

### Genome characterisation

**Figure 1:**
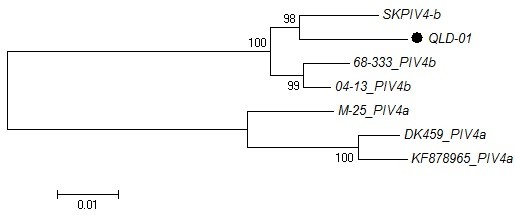
**Phylogenetic tree of concatenated full-genome PIV4 ORFs, including QLD-01.** Maximum likelihood analyses of concatenated NP, P, M, F, HN and L open reading frames of PIV4 isolates AB543337, AB543336, KF483663, JQ241176, EU627591, KF878965, KF908238 using the Tamura-Nei substitution model with 1000 bootstrap replicates. Scale represents estimated nucleotide substitutions per sequence position. This study’s genome is indicated by a filled circle.

Overall QLD-01 sequence similarity to the existing whole PIV4 genomes’ concatenated coding regions showed a similarity ranging from 88.81-97.05%. Phylogenetic analyses confirmed QLD-01 was a divergent member of the 4b subtype clade (Figure 1), while the second PIV4 clustered within the 4a subtype (Figure 1, KF878965). Predicted protein similarities indicated QLD-01’s closest homology to SKPIV4 in most, but not all proteins, with the greatest variation being found in the V protein (91.30-91.74%). No evidence of recombination between QLD-01 and other isolates was found (see Additional files 3, 4, and 5).

Strain QLD-01 and the recently described divergent PIV4a isolate DK(459) shared several structural features identified by Alquezar-Planas *et al.*[9]; an extended C-terminal end and a 13 residue stretch within the globular head of the predicted HN protein, as well as a 57 nt insertion at the 3′ leader non-coding sequence. These features were not unique to the two isolates, and in the case of the HN protein, were more common than not across both genotypes. Conversely, QLD-01 contained the most divergent residue sequence (91.4-95.0%) of the PIV4b genotype within the variable C terminal end (residues 411–451) of the Nucleocapsid protein.

### PCR target conservation

Due to the variant nature of QLD-01, a literature search was undertaken to assess the compatibility of published PCR primer/probe targets with the isolate. Of the six assays evaluated [3,5,15-18], four contained mismatches in their primer/probe sequences, in particular at the 3′ end (Table 1).

**Table 1:** PIV4 PCR oligomer mismatches to isolate QLD-01

**Primer name**	**Sequence (5′-3′)**	**Study**
PI4P+	CTGAACGGTTGCATTCAGG **T**	[3]
PIASB+	AA **C** CAGGGAAACAGAGC **T** C	[3]
LPW 1779	GTGTCTGATCCCATA AGCAG **C**	[5]
PIV-4 Reverse	GCATGTTCTGC **A** TCTCT **G** GA	[16]
PIV 4 Forward	CAAA **T** GATCCACAGCAAAGATT **C**	[18]
PIV 4 Reverse	ATGTGGCCTGTAA **G** GAAAGCA	[18]
PIV 4 Probe	GTATCATCATCTGCCAA **A** TC **G** GCAAT **T** AAACA	[18]

Published PIV4 primer and probe sequences with mismatches to isolate QLD-01 underlined and in bold.

## Discussion

Improvements in sequencing and detection technologies over the past 15 years have led to increasing detection rates of existing, neglected, and unknown pathogens. The existence of PIV4 has been known since 1959; however only recently has PIV4 been more appreciated as a respiratory pathogen in its own right through the use of modern molecular methods [1-5,8]. Modern molecular diagnostics heavily rely on PCR-based techniques. Because of their high specificity, these same methods are susceptible to decreased sensitivity or even false negative results when confronted with even minor changes in target sequences. This limitation is particularly relevant to clinically important pathogens for which little sequence data are available to guide PCR assay development and ongoing evaluation.

The variant PIV4 isolate described in this study is the most divergent of the five available whole genome sequences. Thus it is not surprising that four of the six evaluated published PCR assays contained potentially deleterious mismatches with the isolate. In particular, all four assays contain mismatches at or near their primers’ 3′ ends, which are especially sensitive to incorrect base pairing and would potentially lead to decreased primer binding efficiency, and in conjunction with the other primer mismatches, false negative results. These mismatches illustrate the difficulty in designing sensitive PCR assays based on very limited sequence information. Additionally, the use of commercial assays for which primer sequences are not readily available, such as those used in recent PIV4 epidemiological studies [1,8], should be used with the understanding that there is no capacity to evaluate the assays’ target sequence conservation as new data on emerging variant viral strains becomes available.

Isolate QLD-01 was found in the upper respiratory tract of an adult with ILI but no other known conditions. It was the sole virus detected, despite extensive screening for other known and unknown respiratory pathogens, thus supporting a potential role as the aetiological agent of the subject’s ILI symptoms. Recent studies have reported PIV4 infections associated with both lower and upper respiratory tract symptoms within the hospital setting [1-5,8]. This study provides further evidence of PIV4’s possible involvement in upper respiratory tract infections in otherwise healthy adults within the community setting.

In regard to its genomic structure, QLD-01’s highest similarity was to isolate SKPIV4. However its overall genomic and NP C-terminal end divergence separate it from SKPIV4 and other PIV4b genomes. In other parainfluenza viruses, the NP C terminus binds the protein-associated viral RNA to the RNA polymerase [19], thus QLD-01’s variant C-terminal end may alter the isolate’s viral RNA synthesis kinetics. Isolate QLD-01’s shared genomic features with both its closest PIV4b homologue and the divergent PIV4a raises the possibility of recombination events occurring between the viral subtypes, however no conclusive evidence was observed to support this hypothesis.

## Conclusions

In this study, the utility of a combined RT-PCR and next-generation sequencing approach to identifying novel viral pathogen was demonstrated with the discovery of a variant strain of PIV4. The whole coding region of the variant strain was sequenced and showed that the majority of publically available PIV4 PCR assays contained mismatches when aligned to this variant, which may lead to decreased sensitivity and false negative results, thereby underestimating the prevalence of PIV4.

## Competing interests

The authors declare that they have no competing interests.

## Authors’ contributions

SB: sample sequencing, assay design, genome assembly, data analyses, manuscript drafting and revision. JM, TN, & SBL: project conception, manuscript drafting and revision. GZ: pyrosequencing & data analyses. DW: project conception, data analyses, manuscript drafting and revision. MWD: project conception, manuscript revision. TPS: project conception and supervision, manuscript drafting and revision. All authors read and approved the final manuscript.

## Pre-publication history

The pre-publication history for this paper can be accessed here:

http://www.biomedcentral.com/1471-2334/14/275/prepub

## Supplementary Material

Additional file 1 **Genome walking primers.** PCR primers used to amplify and sequence PIV4 strain QLD-01. All sequences are shown in the 5′-3′ orientation. Click here for file

Additional file 2 **454 GS FLX QLD-01 reads.** Table showing positions of the original 32 reads mapped to the SKPIV4 genome. Click here for file

Additional file 3 **Recombination analyses of PIV4 genomes; 1719 nt window.** RAT analyses of PIV4 genomes using a 1719 nt scanning window. QLD-01 is used as the reference genome. Nucleotide position is shown on the x-axis, and relative percentage identity is shown on the y-axis. Click here for file

Additional file 4 **Recombination analyses of PIV4 genomes; 800 nt window.** RAT analyses of PIV4 genomes using a 800 nt scanning window. QLD-01 is used as the reference genome. Nucleotide position is shown on the x-axis, and relative percentage identity is shown on the y-axis. Click here for file

Additional file 5 **Recombination analyses of PIV4 genomes; 400 nt window.** RAT analyses of PIV4 genomes using a 400 nt scanning window. QLD-01 is used as the reference genome. Nucleotide position is shown on the x-axis, and relative percentage identity is shown on the y-axis. Click here for file
