# Supplementary figures and images for: Detection of a divergent Parainfluenza 4 virus in an adult patient with influenza like illness using next-generation sequencing
Source: BMC Infect Dis. 2014 May 19;14:275. doi: 10.1186/1471-2334-14-275 (PMC4038074; doi:10.1186/1471-2334-14-275)

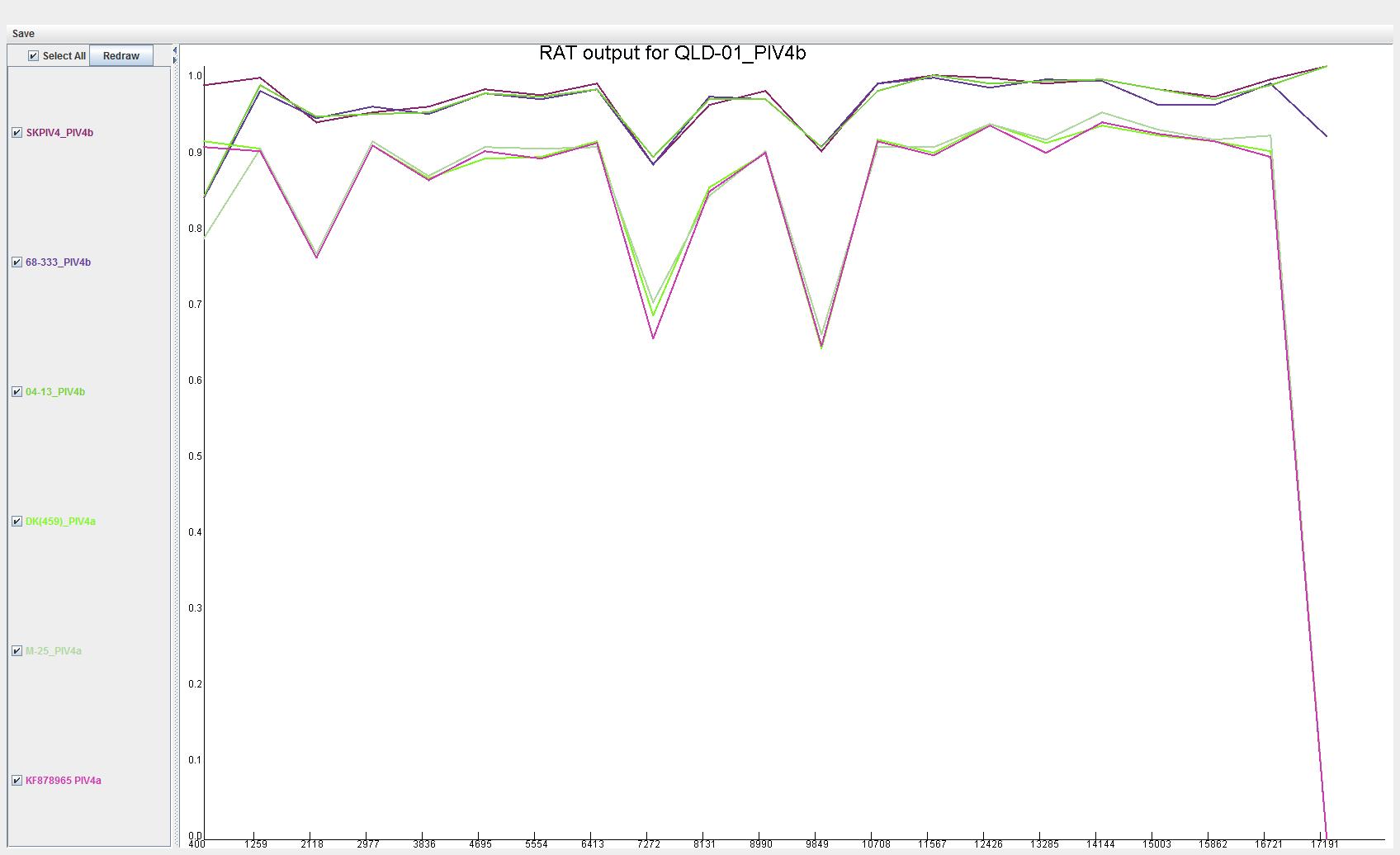

Supplement: Additional file 3 — Recombination analyses of PIV4 genomes; 1719 nt window. RAT analyses of PIV4 genomes using a 1719 nt scanning window. QLD-01 is used as the reference genome. Nucleotide position is shown on the x-axis, and relative percentage identity is shown on the y-axis. [file 1471-2334-14-275-S3.jpeg]

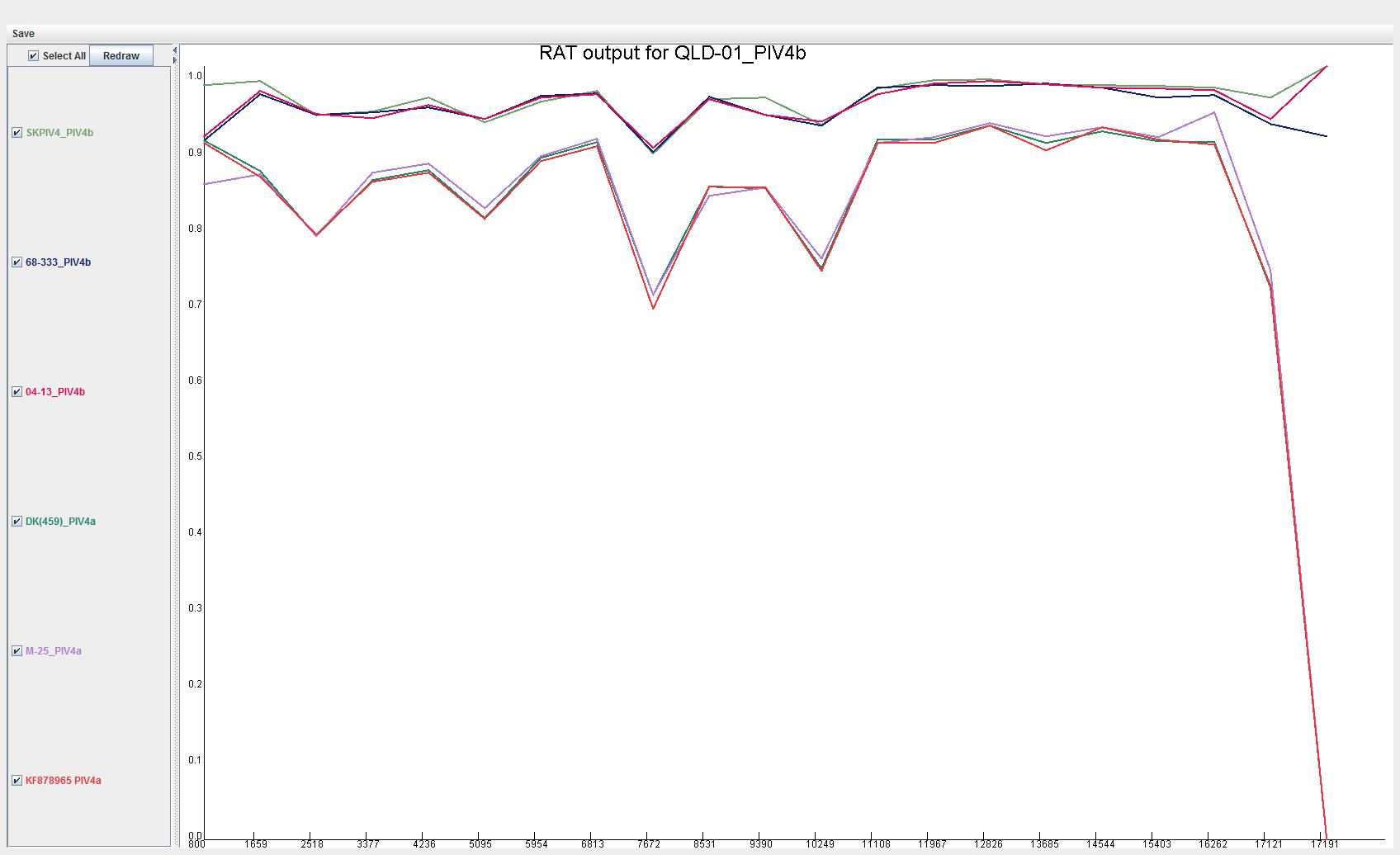

Supplement: Additional file 4 — Recombination analyses of PIV4 genomes; 800 nt window. RAT analyses of PIV4 genomes using a 800 nt scanning window. QLD-01 is used as the reference genome. Nucleotide position is shown on the x-axis, and relative percentage identity is shown on the y-axis. [file 1471-2334-14-275-S4.jpeg]

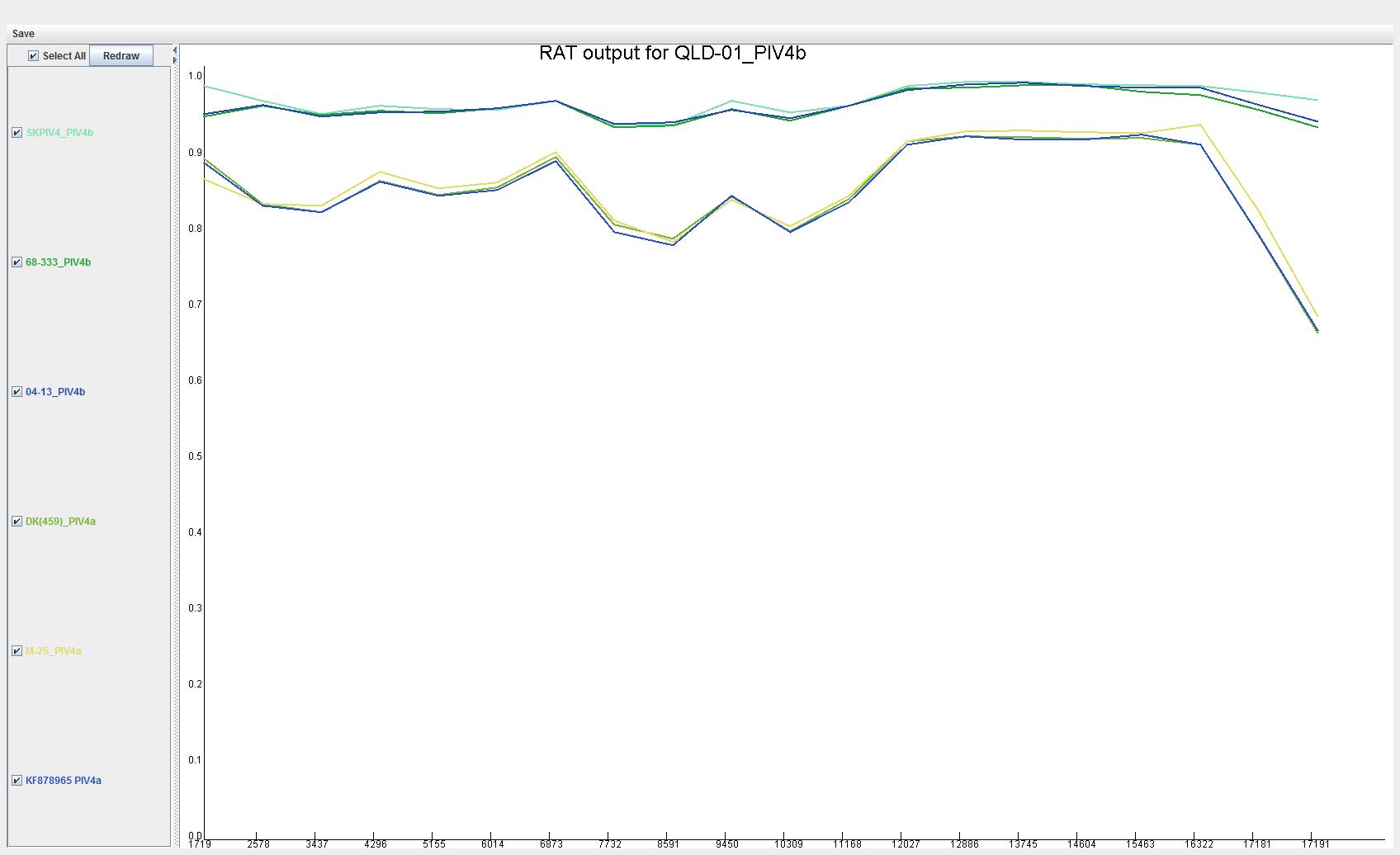

Supplement: Additional file 5 — Recombination analyses of PIV4 genomes; 400 nt window. RAT analyses of PIV4 genomes using a 400 nt scanning window. QLD-01 is used as the reference genome. Nucleotide position is shown on the x-axis, and relative percentage identity is shown on the y-axis. [file 1471-2334-14-275-S5.jpeg]
